# *Eimeria tenella Eimeria*-specific protein that interacts with apical membrane antigen 1 (*Et*AMA1) is involved in host cell invasion

**DOI:** 10.1186/s13071-020-04229-5

**Published:** 2020-07-25

**Authors:** Cong Li, Qiping Zhao, Shunhai Zhu, Qingjie Wang, Haixia Wang, Shuilan Yu, Yu Yu, Shashan Liang, Huanzhi Zhao, Bing Huang, Hui Dong, Hongyu Han

**Affiliations:** 1 Shanghai Veterinary Research Institute, Chinese Academy of Agricultural Sciences, Key Laboratory of Animal Parasitology of Ministry of Agriculture, Minhang, Shanghai 200241 PR China; 2 College of Life and Environment Sciences, Shanghai Normal University, Shanghai, 200234 China

**Keywords:** *Eimeria tenella*, Apical membrane antigen 1, *Eimeria*-specific protein

## Abstract

**Background:**

Avian coccidiosis is a widespread, economically significant disease of poultry, caused by several *Eimeria* species. These parasites have complex and diverse life-cycles that require invasion of their host cells. This is mediated by various proteins secreted from apical secretory organelles. Apical membrane antigen 1 (AMA1), which is released from micronemes and is conserved across all apicomplexans, plays a central role in the host cell invasion. In a previous study, some putative *Et*AMA1-interacting proteins of *E. tenella* were screened. In this study, we characterized one putative *Et*AMA1-interacting protein, *E. tenella Eimeria* -specific protein (*Et*Esp).

**Methods:**

Bimolecular fluorescence complementation (BiFC) and glutathione S-transferase (GST) fusion protein pull-down (GST pull-down) were used to confirm the interaction between *Et*AMA1 and *Et*Esp *in vivo* and *in vitro.* The expression of *Et*Esp was analyzed in different developmental stages of *E. tenella* with quantitative PCR and western blotting. The secretion of *Et*Esp protein was tested with staurosporine when sporozoites were incubated in complete medium at 41 °C. The localization of *Et*Esp was analyzed with an immunofluorescence assay (IFA). An *in vitro* invasion inhibition assay was conducted to assess the ability of antibodies against *Et*Esp to inhibit cell invasion by *E. tenella* sporozoites.

**Results:**

The interaction between *Et*AMA1 and *Et*Esp was confirmed with BiFC and by GST pull-down. Our results show that *Et*Esp is differentially expressed during distinct phases of the parasite life-cycle. IFA showed that the *Et*Esp protein is mainly distributed on the parasite surface, and that the expression of this protein increases during the development of the parasite in the host cells. Using staurosporine, we showed that *Et*Esp is a secreted protein, but not from micronemes. In inhibition tests, a polyclonal anti-r*Et*Esp antibody attenuated the capacity of *E. tenella* to invade host cells.

**Conclusion:**

In this study, we show that *Et*Esp interacts with *Et*AMA1 and that the protein is secreted protein, but not from micronemes. The protein participates in sporozoite invasion of host cells and is maybe involved in the growth of the parasite. These data have implications for the use of *Et*AMA1 or *Et*AMA1-interacting proteins as targets in intervention strategies against avian coccidiosis.
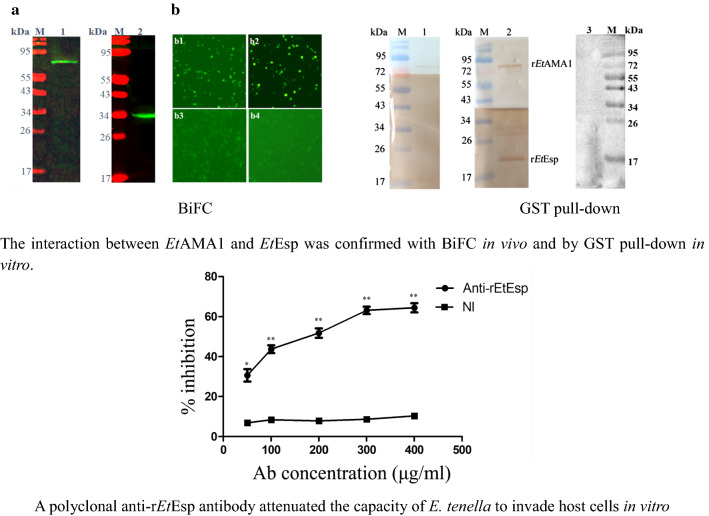

## Background

Avian coccidiosis is a widespread, economically significant disease of poultry that results in annual global economic losses of approximately $2.4 billion, including both production losses and disease prevention and treatment costs [1]. It is an enteric disease caused by several species of the protozoan genus *Eimeria*, predominantly *E. acervulina, E. brunetti*, *E. maxima*, *E. mitis, E. necatrix*, *E. praecox* and *E. tenella* [2]. Of these, *E. tenella* is one of the species causing hemorrhagic pathologies and high mortality. *Eimeria* spp. belong to the phylum Apicomplexa, which includes important pathogens of humans and domestic animals, such as the causative agents of malaria (*Plasmodium* spp.), toxoplasmosis (*Toxoplasma gondii*), babesiosis (*Babesia* spp.), and coccidiosis (*Eimeria* spp.). Most apicomplexans are obligate intracellular parasites and are characterized by their apical complexes of specialized secretory organelles (micronemes, rhoptries and dense granules) [3]. They use actin-based motility coupled to regulated protein secretion from their apical organelles to actively invade host cells [4]. These parasites have complex and diverse life-cycles that involve the invasion of many different cell types, including erythrocytes, lymphocytes, macrophages, and digestive-tract cells. Despite the diversity of their target host cells, they maintain a highly conserved mechanism for this active invasion process [5].

The host-cell invasion mechanism involves four steps, i.e. attachment, apical reorientation, moving junction formation, and the formation of a protective parasitophorous vacuole. Each invasion step is mediated by various proteins, which are secreted from apical secretory organelles [6]. Apical membrane antigen 1 (AMA1), a type I transmembrane protein, is one of a number of proteins released from micronemes that are conserved across all apicomplexans. It is known to play several important roles during host-cell penetration [7]. For instance, previous reports have shown that antibodies against AMA1 or small specific AMA1-binding peptides inhibit the invasion of host cells by *Toxoplasma* spp., *E. tenella*, *Babesia* spp., *Neospora* spp. and *Plasmodium* spp. [3, 8–11]. AMA1 is also a long-standing effective candidate vaccine for some apicomplexans, including *N. caninum*, *T. gondii* and *Plasmodium* spp. [11–13]. In *Toxoplasma* spp. and *Plasmodium* spp., AMA1 is reportedly involved in apical reorientation [14], host-cell attachment [7, 15], invasion and establishment of the moving-junction [16], and the provision of a signal that initiates intracellular replication [17].

In contrast to the functions of AMA1 in other apicomplexan parasites, there are only a few reports of this conserved protein in *Eimeria* spp. In a previous *in vitro* study, AMA1 antibodies or specific *Et*AMA1-binding peptides inhibited the invasion of host cells by *E. tenella* sporozoites [10, 18]. *Et*AMA1 also partially protected host cells against homologous challenge with *E. tenella* when used as a recombinant protein vaccine and against heterologous challenge with *E. maxima* when the AMA1 protein from *E. maxima* was expressed as a live vectored vaccine [19]. Although AMA1 plays an important role in host-cell invasion by *E. tenella* sporozoites, its precise functions are unknown.

Proteins perform a vast number of cellular functions when they interact with one or multiple binding partners. Protein-protein interactions are essential in the mediation of almost all cellular processes, including replication, transcription, translation and signal transduction [20]. The biochemical analysis of protein complexes and the identification of their components have been fundamental to our understanding of their biological functions in cells [21].

To understand the precise functions of *Et*AMA1 during host-cell invasion, we screened *Et*AMA1-interacting proteins with a yeast two-hybrid system and identified 14 putative *Et*AMA1-interacting proteins in a previous study [22]. *E tenella Eimeria*-specific protein (*Et*Esp) (GenBank: JZ905773) is one of these putative interacting proteins. In this study, we cloned and characterized *Et*Esp. We systematically analyzed its interaction with *Et*AMA1 using bimolecular fluorescence complementation (BiFC) *in vivo* and a glutathione S-transferase (GST) pull-down assay *in vitro*. Our results show that the *Et*Esp is secreted protein, but not from micronemes, interacts with *Et*AMA1, and is involved in the invasion of host cells by *E. tenella* sporozoites.

## Methods

### Parasite collection

*Eimeria tenella* was obtained from the Key Laboratory of Animal Parasitology of the Ministry of Agriculture, Shanghai Veterinary Research Institute, the Chinese Academy of Agricultural Sciences, Shanghai, China. The parasites were maintained and propagated by passage through coccidia-free, 2-week-old chickens, as previously described [23]. Coccidia-free 14-day-old chickens were inoculated with 1 × 10^4^ sporulated oocysts of *E. tenella*. Unsporulated oocysts (UO) were collected from infected chicken ceca at 7 days post-infection. Sporulated oocysts (SO) were derived from UO that had undergone sporulation in 2% potassium dichromate at a temperature of 28–30 °C for 72–120 h, under forced aeration with a suitable pump. When more than 90% of the oocysts had sporulated, the oocysts were collected and purified. The sporozoites (Spz) were purified from cleaned SO with *in vitro* excystation [24]. Second-generation merozoites (sMrz) were isolated from infected chicken ceca at 115 h post-inoculation, as described previously [25]. All parasites were collected and frozen in liquid nitrogen.

Chickens and rabbits were fed and used according to a protocol approved by the Animal Care and Use Committee of the Shanghai Veterinary Research Institute, Chinese Academy of Agricultural Sciences.

The chicken embryo fibroblast cell line, DF-1, a derivative of the East Lansing Line (ELL-0) [10], was used for BiFC and *in vitro* infection experiments.

### Molecular cloning and sequence analysis of *E. tenella*-specific protein

Total RNA was extracted from *E. tenella* sporozoites with TRIzol reagent (Invitrogen, Carlsbad, CA, USA). GeneRacer™ primers (GR5P and GR5N) were provided for the random amplification of PCR ends (RACE) in the GeneRacer™ Kit (Invitrogen) and gene-specific primers (GS5P and GS5N) were designed based on the expressed sequence tag (EST) sequence (GenBank: JZ905773) which is 790 bp in length and contains a poly(A) at the 3’-end (Additional file 1: Table S1). The 5’-end of this gene was determined according to the manufacturer’s protocol. The PCR-amplified fragment was then ligated into the pGEM-T Easy Vector (Promega, Madison, WI, USA) and used to transform competent *E. coli* TOP10 cells. After PCR identification, the plasmid DNA was sequenced. After the resulting sequence was assembled and aligned with the original EST sequence, the full-length cDNA sequence of the gene was determined and submitted to the National Center for Biotechnology Information (NCBI) GenBank under the accession number MN161778). The full-length *Et*Esp cDNA sequence was used in a BLAST search of the GenBank database (http://www.ncbi.nlm.nih.gov/BLAST/) and the *E. tenella* genome database (http://www.genedb.org/Homepage/Etenella). The deduced amino acid sequence was obtained with the ORF Finder tool at NCBI. The molecular mass and theoretical isoelectric point were calculated with ProtParam tools (http://web.expasy.org/protparam/). The signal peptide sequence was identified with the SignalP 4.1 server (http://www.cbs.dtu.dk/services/SignalP/), and transmembrane regions were predicted with the TMHMM server v. 2.0 (http://www.cbs.dtu.dk/services/TMHMM/). Protein motifs were scanned with Motif Scan (http://myhits.isb-sib.ch/cgi-bin/motif_scan).

### Recombinant protein expression and polyclonal anti-r*Et*Esp serum

The *Et*Esp open reading frame (ORF) cDNA was amplified with PCR using primers *Et*Esp-UP and *Et*Esp-LP (Additional file 1: Table S1), which contained *Bam*HI and *Xho*I restriction sites, respectively. The PCR fragment was then ligated into the prokaryotic expression vector pET28a(+) digested with the same restriction endonucleases, to construct the recombinant expression plasmid pET-*Et*Esp. The recombinant protein His-*Et*Esp (r*Et*Esp) was expressed in *Escherichia coli* BL21 cells at 37 °C with 1 mM isopropyl-thio-α-d-galactoside. The cell pellet was lysed with sonication and digested with 10 µg/ml lysozyme (Sigma-Aldrich, St. Louis, MO, USA). The lysate was then analyzed with 12% SDS-PAGE to confirm that the recombinant protein was present as a soluble protein or inclusion bodies. r*Et*Esp was purified with His·Bind® Resin (Merck, Darmstadt, Germany) and its concentration measured with a BCA Protein Assay Kit (Beyotime, Haimen, China).

Two 2-month-old male rabbits were inoculated with 200 μg of purified r*Et*Esp emulsified in Freund’s complete adjuvant (Sigma-Aldrich). After 14 days, a booster of 200 μg of purified r*Et*Esp in Freund’s incomplete adjuvant (Sigma-Aldrich) was administered, followed by a second and third booster on days 28 and 42. One week after the final booster, the rabbit serum was collected and stored at − 20 °C until use.

### Analysis of *Et*Esp transcript levels with real-time quantitative PCR (qPCR)

The expression profiles of *Et*Esp mRNA were examined in four developmental stages of *E. tenella* (UO, SO, Spz and sMrz) with qPCR. cDNA samples were synthesized from DNaseI-treated total RNAs of the *E. tenella* developmental stages using SuperScript™ II Reverse Transcriptase (Invitrogen) and random pd(N)6 primer. The housekeeping *18S* rRNA gene was used as the internal control. The primers used to amplify the *Et*Esp cDNA (*Et*Esp-SP and *Et*Esp-AP) and the *18S* rRNA gene (18S-SP and 18S-AP) were designed with Primer3 v. 0.4.0 (http://bioinfo.ut.ee/primer3-0.4.0/) (Additional file 1: Table S1). qPCR was performed with the StepOnePlus™ Real-Time PCR System using the SYBR® Premix Ex Taq™ II kit (Takara, Tokoyo, Japan). All experiments were performed twice, with separate biological replicates. In each experiment, the reactions were performed in triplicate. A dilution series of cDNA templates of the sporozoites was used to establish standard curves, and all standard curves had correlation coefficients of *R*^2^ > 0.99. The comparative 2^−ΔΔCq^ method was used to analyze the relative levels of gene expression.

### SDS-PAGE and western blotting

Protein samples were prepared from the four *E. tenella* developmental stages (UO, SO, Spz and sMrz), and from DF-1 cells transfected with the recombinant plasmids, for western blotting. The protein concentrations were determined with a BCA Protein Assay Kit (Beyotime). The purified r*Et*Esp and protein lysates were separated with SDS-PAGE and transferred electrophoretically to polyvinylidene difluoride membranes. Rabbit antiserum (1:100) against sporozoite proteins, previously produced in our laboratory [26], a rabbit anti-r*Et*Esp antibody (1:100), a mouse monoclonal anti-α-tubulin antibody (1:1000) (Sigma-Aldrich), and a monoclonal anti-His antibody (1:1000) were used as the primary antibodies to detect r*Et*Esp or native *Et*Esp. Naïve rabbit serum (1:100) was used as the negative control. IRDye-800CW-labelled goat anti-rabbit IgG antibody (1:25,000) and IRDye-680RD-labeled donkey anti-mouse IgG antibody (1:25,000; LI-Cor, Lincoln, NE, USA) were used as the secondary antibodies. The IRDyes were detected with the Odyssey Infrared Imaging System (LI-Cor).

### BiFC assay

The ORF fragments of *Et*Esp and the *Et*AMA1 ectodomain, with no stop codon, were amplified from the first-strand cDNA with two pairs of primers (Bf*Et*Esp-UP/Bf*Et*Esp-LP and Bf*Et*AMA1-UP/Bf*Et*AMA1-LP, respectively), which contained *Eco*RI and *Bgl*II restriction sites (*Et*Esp) or *Eco*RI and *Xho*II restriction sites (*Et*AMA1). The fragments were then digested with the appropriate restriction enzymes and ligated into the pBiFC-VN155 and pBiFC-VC155 vectors digested with the same enzymes, respectively, to construct the recombinant plasmids pBiFC-VN155-*Et*Esp and pBiFC-VC155-*Et*AMA1, respectively. Before the BiFC assay, the uptake of the expression vectors by the cells was confirmed. DF-1 cells were transfected with the recombinant plasmid pBiFC-VN155-*Et*Esp or pBiFC-VC155-*Et*AMA1 using Lipofectamine™ 2000 Transfection Reagent (Invitrogen), according to the manufacturer’s instructions. At 48 h after transfection, the cells were harvested, and the proteins were extracted with RIPA Lysis Buffer (Beyotime). Western blots were probed with rabbit anti-r*Et*Esp antibody and rabbit anti-r*Et*AMA1 antibody, which were previously produced in our laboratory [10]. After confirmation that the cells had expressed the two constructs, DF-1 cells were cotransfected with pBiFC-VN155-*Et*Esp and pBiFC-VC155-*Et*AMA1. DF-1 cells were also cotransfected with pBiFC-bJunVN55 (I152L) and pBiFC-bFosVC155 or pBiFC-VC155-*Et*AMA1 and pBiFC-VN155 empty vector, or pBiFC-VN155-*Et*Esp and pBiFC-VC155 empty vector as the positive or negative control, respectively. The DF-1 cells were observed with fluorescence microscopy 24 h after transfection with the different constructs.

### GST pull-down

To confirm the interaction between *Et*AMA1 and *Et*Esp317 *in vitro*, a GST pull-down assay was performed with the Pierce™ GST Protein Interaction Pull-Down Kit (Thermo Fisher Scientific, Waltham, MA, USA), according to the manufacturer’s instructions. The recombinant plasmid pGEX-6P-*Et*AMA1 was previously constructed in our laboratory [10]. The expression of the recombinant protein GST-*Et*AMA1 was induced and the protein purified with GST resin for use as the bait protein. The ORF of *Et*Esp was inserted into the pET-28a vector to express the recombinant protein His-*Et*Esp (r*Et*Esp) as the prey protein. GST-*Et*AMA1 was incubated with equilibrated glutathione-agarose to immobilize the bait protein. r*Et*Esp was then added to the glutathione-agarose and incubated with the bait protein. The bait and prey proteins were eluted from the glutathione-agarose. *E. coli* BL21 cells were transformed with recombinant plasmid pET-*Et*MIC2, constructed previously in our laboratory [27], to express the recombinant protein His-*Et*MIC2 as the negative control. Another, r*Et*Esp was loaded in an empty glutathione-agarose column as the negative. All the proteins were then resolved with 12% SDS-PAGE and detected with western blotting using the appropriate antibodies, as described above.

### Assay of *Et*Esp secretion

Freshly excysted sporozoites (4 × 10^6^) were incubated in 100 μl of complete medium (CM; Dulbecco’s modified Eagle’s medium (DMEM) supplemented with 10% fetal bovine serum (FBS), 100 U/ml penicillin/streptomycin, 2 mM l-glutamine) for 2 h at 41 °C under 5% CO_2_ for the secretion experiments. They were then incubated with 5, 10 or 20 μM staurosporine (Sigma-Aldrich; dissolved in dimethylsulfoxide (DMSO)) or an appropriate volume of carrier DMSO, as described previously [28]. The secretion of *Et*MIC2 and *Et*GRA(*Tg*GRA7 homologous protein) was used as the control. The sporozoites were then pelleted by centrifugation for 10 min at 6000× *g*. The supernatants and sporozoites were recovered and analyzed with western blotting using a rabbit anti-r*Et*Esp antibody and rabbit anti-r*Et*MIC2 antibody generated previously in our laboratory [27] and mouse anti-*Tg*GAR7 antibody which had been generated previously in another laboratory at Shanghai Veterinary Research Institute, Chinese Academy of Agricultural Sciences.

### Immunofluorescence staining of parasites

Purified differentially developed parasites (Spz, sporocysts [Sporo], and sMrz) were transferred to glass slides and air-dried, as previously described [10, 29]. Freshly purified sporozoites were used to infect DF-1 cells after incubation in CM for 2 h at 41°C. At different time points after infection, the DF-1 cells were collected, washed, transferred to glass slides, and air-dried. The slides were then fixed in 2% paraformaldehyde in phosphate-buffered saline (PBS) and placed in 1% Triton X-100 in PBS for 15 min to increase their permeability. Non-permeabilized sporozoites and sporocysts were used as a control. The slides were blocked with PBS containing 2% (w/v) bovine serum albumin for overnight at 4 °C. A rabbit anti-r*Et*Esp antibody (1:100) was added and the cells were incubated for 1 h at 37 °C. A 1:500 dilution of fluorescein isothiocyanate (FITC)-conjugated goat anti-rabbit IgG antibody (Sigma-Aldrich) was then added and the cells incubated for 1 h at 37 °C. The cell nuclei were stained by incubation in 10 μg/ml 4′,6-diamidino-2-phenylindole (Beyotime) at room temperature for 10 min. After each step, the slides were washed three times for 10 min each with PBS containing 0.05% Tween 20. The slides were finally mounted with 50 μl of Fluoromount Aqueous Mounting Medium (Sigma-Aldrich) before observation with a fluorescence microscope (Olympus, Tokyo, Japan). At the same time, we performed the co-localization of *Et*Esp and *Et*AMA1 in sporozoites. Purified sporozoites were treated with mouse anti-r*Et*AMA1 antibody (1:100) and rabbit anti-r*Et*Esp antibody (1:100), then goat anti-rabbit IgG fluorescein isothiocyanate (FITC)-conjugated antibody (1:500) and goat anti-mouse IgG cyanine (Cy3)-conjugated antibody (1:500; Sigma-Aldrich) were used as secondary antibodies.

### Invasion inhibition assay *in vitro*

The invasion inhibition assay was based on previous reports of the invasion of DF-1 cells by *E. tenella* sporozoites. Antibodies were purified with Protein A + G Agarose (Beyotime). DF-1 cells (2 × 10^5^ cells per well) were cultured in 24-well plates (Corning, UN, USA) in CM for 24 h at 37 °C under 5% CO_2_. The freshly purified sporozoites were counted and labeled with carboxyfluorescein diacetate succinimidyl ester (Beyotime). The labeled sporozoites were incubated at 37 °C with 50, 100, 200, 300 or 400 μg/ml purified IgG directed against r*Et*Esp for 2 h. The same quantity of IgG from naïve rabbit serum (Sigma-Aldrich) was used as the negative control, and an equivalent volume of PBS as the normal control. After they were washed twice with sterile PBS, DF-1 cells (10^5^/well) were infected with the labeled sporozoites (10^5^/well) in 24-well plates and cultured for 16 h at 41 °C under 5% CO_2_. The cells were then collected and analyzed with flow cytometry on a Cytomics™ FC 500 (Beckman Coulter, Indianapolis, IN, USA). The controls were uninfected DF-1 cells. The infected cells, uninfected cells, and free sporozoites were gated using the CXP software (Beckman Coulter) to count the infected (labeled sporozoites) and uninfected (fluorescence-free) cells. All assays were performed in triplicate. The percentage of infected cells in the presence or absence of an anti-r*Et*Esp polyclonal antibody were used to calculate the inhibition rates, as previously described [10].

## Results

### Cloning and sequence analysis of full-length *Et*Esp cDNA

**Fig. 1:**
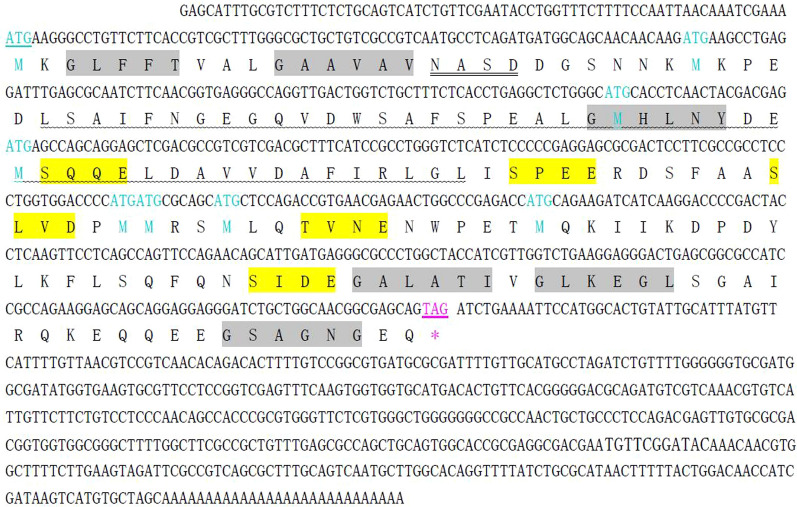
Nucleotide sequence of the full-length cDNA of *Et*Esp and the deduced amino acid sequence. Start and stop codons are underlined. One putative intein DOD-type homing endonuclease domain is shown with wavy underlining. A putative N-glycosylation site has a double line. Five putative casein kinase II phosphorylation sites are shown in yellow. Six putative N-myristoylation sites are shown in gray

The 1108 bp full-length cDNA of *Et*Esp was obtained with RACE. The sequence analysis showed that the full-length cDNA included a 5’-untranslated region (UTR) of 70 bp, a 3’-UTR of 542 bp with a poly(A) tail, and an ORF of 501 bp, which encoded 166 amino acids with a calculated molecular weight of 18.1 kDa and a theoretical isoelectric point of 4.2 (Fig. 1). Analysis of the amino acid sequence showed a signal peptide of 19 amino acids at the N-terminus and no transmembrane region. Searches in the Motif Database and the Conserved Domain Database revealed the presence of 1 N-glycosylation site, 5 casein kinase II phosphorylation sites, 6 N-myristoylation sites, 1 tyrosine kinase phosphorylation site, 1 intein DOD-type homing endonuclease domain, and no conserved domains (Fig. 1). A BLAST search of the *E. tenella* genome database showed that the ORF sequence shared 100% sequence identity with ETH_00016590, which encodes an *Eimeria*-specific protein, on supercontig Eth_scaff124: 9216–10141.

The amino acid sequence shared 100% homology with the *E. tenella Eimeria*-specific protein (XP_013228647.1) and 92% (152/170) identity with the *E. necatrix Eimeria*-specific protein (XP_013435139.1) in NCBI. Therefore, this gene was designated *Et*Esp and submitted to the GenBank database under the accession number MN161778. It also shared 68% (106/157) amino acid identity with *E. brunetti* conserved hypothetical protein (CDJ53027.1), 63% (108/172) identity with *E. praecox* conserved hypothetical protein (CDI81636.1), 74% (97/131) identity with *E. maxima* conserved hypothetical protein (XP_013336310.1), and 73% (91/124) identity with *E. acervulina* conserved hypothetical protein (XP_013248166.1). Also, this protein is not found in other apicomplexan parasites. These results show that the protein is conserved in *Eimeria* spp.

### Expression and characterization of recombinant *Et*Esp

**Fig. 2:**
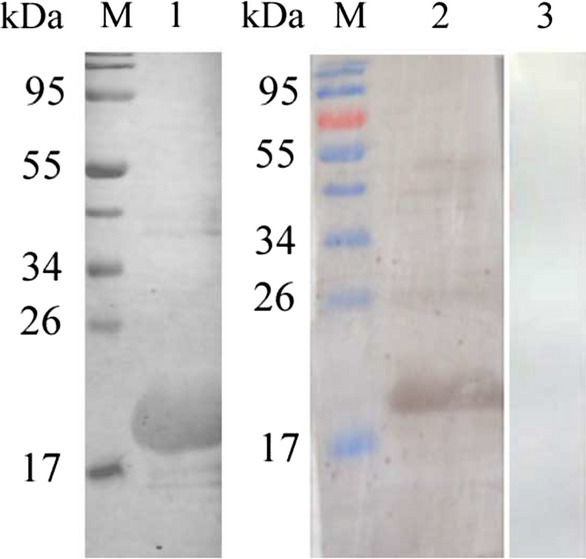
Immunogenicity of r*Et*Esp. r*Et*Esp protein was subjected to western blotting. Lane M: protein marker; Lane 1: anti-His-tag monoclonal antibody as the primary antibody; Lane 2: rabbit serum against sporozoites as the primary antibody; Lane 3: naïve rabbit IgG as the primary antibody

r*Et*Esp was expressed as a His6-tagged fusion protein. SDS-PAGE showed that r*Et*Esp was mainly present in the soluble fraction of the bacterial lysate. After the purification of r*Et*Esp with Ni-NTA chromatography, a protein of approximately 21 kDa was observed with SDS-PAGE. Because 3 kDa of the fusion protein was derived from the vector, the predicted molecular mass of *Et*Esp was about 18.1 kDa. Western blotting showed that purified r*Et*Esp was recognized by rabbit serum directed against sporozoites and by a monoclonal anti-His6 antibody. Naïve rabbit serum failed to recognize any protein corresponding to the expected size of r*Et*Esp (Fig. 2). These results indicate that r*Et*Esp was recognized specifically by rabbit serum directed against a soluble sporozoite protein and by a monoclonal anti-His antibody.

### *Et*Esp mRNA and protein expression at different developmental stages of *E. tenella*

**Fig. 3:**
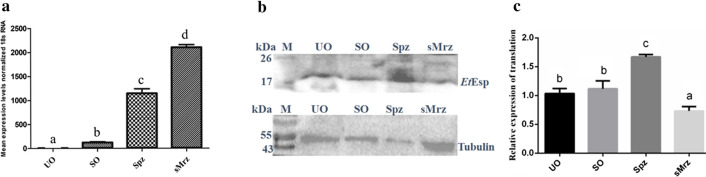
*Et*Esp expression at different developmental stages of *E. tenella*. **a** qPCR of *Et*Esp at different developmental stages of *E. tenella*. Bars with different letters indicate significant differences (*P* < 0.05) and the error bars indicate standard deviations. **b** Western blot showing *Et*Esp at different developmental stages, probed with rabbit anti-r*Et*Esp serum or mouse monoclonal anti-α-tubulin antibody. **c** The densitometric intensity of western blot images was analyzed using ImageJ software. Bars with different letters indicate significant differences (*P* < 0.05) and the error bars indicate standard deviations. *Abbreviations*: UO, unsporulated oocysts; SO, sporulated oocysts; Spz, sporozoites; sMrz, second-generation merozoites

qPCR was used to analyze the UO, SO, Spz, and sMrz stages of *E. tenella* for the presence of *Et*Esp mRNA. The levels of *Et*Esp mRNA were much higher in the sMrz stage than in the other three stages, and *Et*Esp mRNA was almost undetectable in UO (Fig. 3a).

The expression of *Et*Esp in the 4 developmental stages was also determined with immunoblotting using rabbit antiserum against r*Et*Esp. A monoclonal anti-α-tubulin antibody was used as the control. Western blotting showed that the anti-r*Et*Esp antibody reacted with a band of approximately 18 kDa in the parasite lysates prepared from the four different developmental stages of *E. tenella.* The expression levels of *Et*Esp were higher in sporozoites than in other three stages (Fig. 3b, c).

### Confirmation of the interaction between *Et*AMA1 and *Et*Esp

**Fig. 4:**
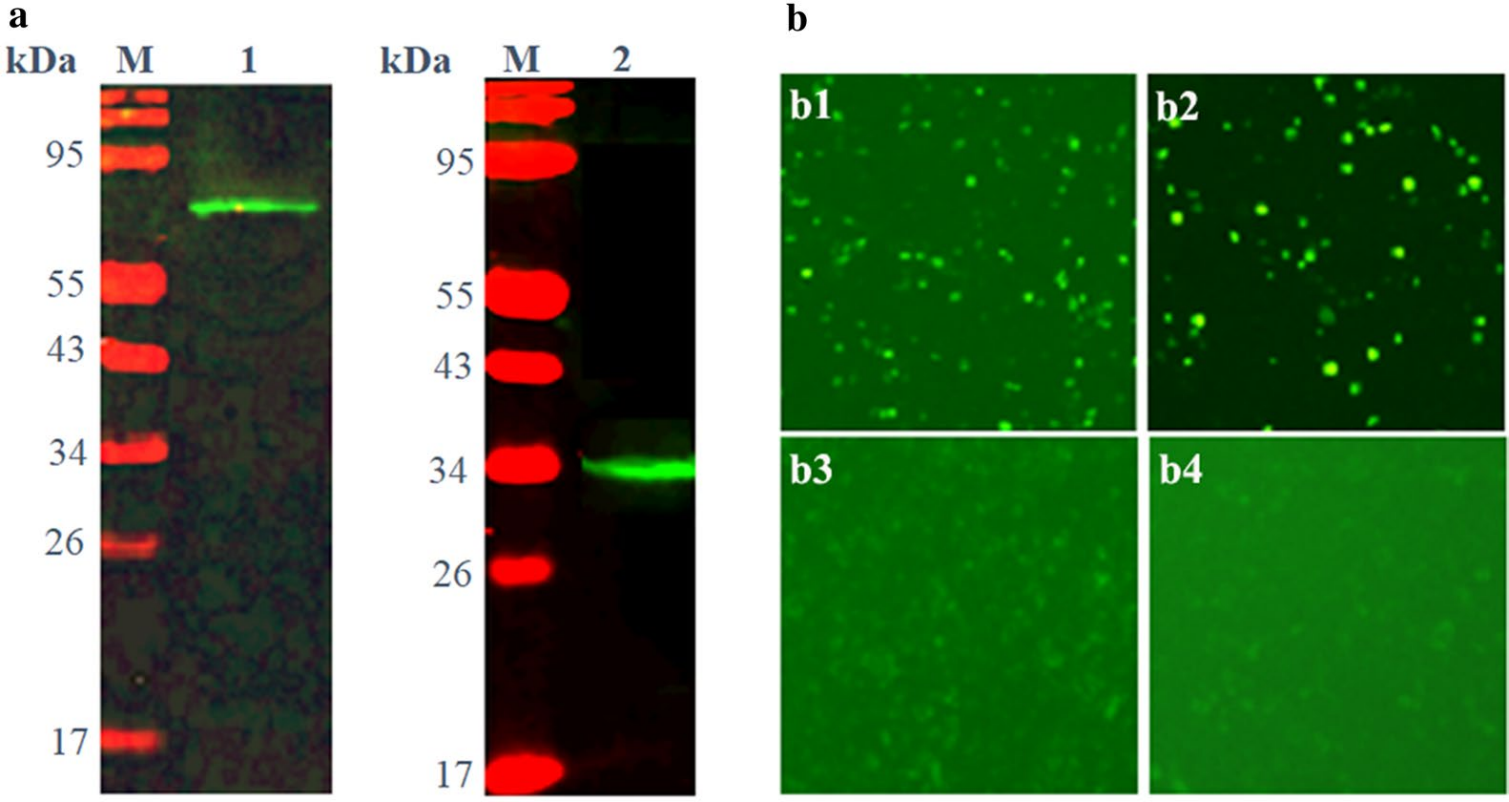
Interaction between *Et*AMA1 and *Et*Esp in DF-1 cells assessed with BiFC. **a** DF-1 cells were transfected with VC155-*Et*AMA1 and VN155-*Et*Esp and the cellular lysates were analyzed with immunoblotting using antisera against *Et*AMA1 and *Et*Esp. Lane 1: anti-r *Et*AMA1 antibody; Lane 2, anti-r *Et*Esp antibody. **b** BiFC was performed. **b1** DF-1 cells were co-transfected with VC155-*Et*AMA1 and VN155-*Et*Esp. **b2** DF-1 cells were co-transfected with positive controls bFos and bJun. **b3** DF-1 cells were co-transfected with pBiFC-VC155-*Et*AMA1 and pBiFC-VN155 empty vector. **b4** DF-1 cells were co-transfected with pBiFC-VN155-*Et*Esp and pBiFC-VC155 empty vector

To characterize the interaction between *Et*AMA1 and *Et*Esp *in vivo*, a BiFC assay was performed. For the BiFC assay, fragments of the *Et*Esp ORF and the *Et*AMA1 ectodomain sequence were cloned into the plasmids pBiFC-VN155 and pBiFC-VC155, respectively, to generate the constructs pBiFC-VN155-*Et*Esp and pBiFC-VC155-*Et*AMA1, respectively. The total proteins were extracted from DF-1 cells transfected separately with one or the other construct. Western blotting showed that the two constructs were expressed individually in the DF-1 cells at 48 h after transfection (Fig. 4a). Strong green fluorescence was observed in DF-1 cells 48 h after they were co-transfected with both constructs. Green fluorescence was also observed in the positive control. However, there was no visible fluorescence in the DF-1 cells co-transfected with pBiFC-VC155-*Et*AMA1 and pBiFC-VN155 empty vector or pBiFC-VN155-*Et*Esp and pBiFC-VC155 empty vector. These results indicate that *Et*Esp interacts with *Et*AMA1 in cells (Fig. 4b).

### GST pull-down

**Fig. 5:**
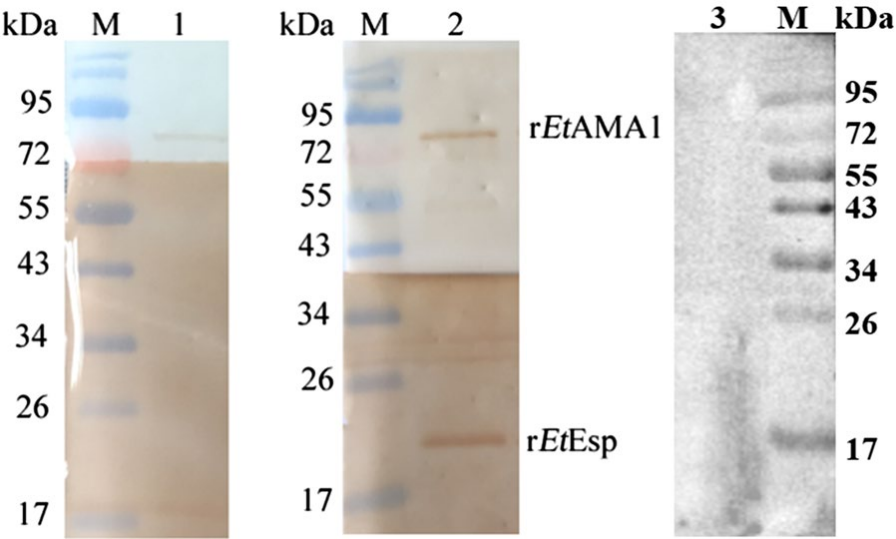
*In vitro* pull-down assay between *Et*AMA1 and *Et*Esp. Lane M: protein marker; Lane 1: r*Et*MIC2 protein incubated with r*Et*Esp, detected with anti-r*Et*AMA1 and anti-r*Et*MIC2 as a negative control; Lane 2: r*Et*AMA1 protein incubated with r*Et*Esp, detected with anti-r*Et*AMA1 and anti-r*Et*Esp; Lane 3: r*Et*Esp loaded in an empty glutathione-agarose column, detected with anti-r*Et*Esp as a negative control

To confirm the interaction between *Et*AMA1 and *Et*Esp *in vitro*, a GST pull-down assay was performed. GST-*Et*AMA1 and His-*Et*Esp were expressed individually in *E. coli* and purified. GST-*Et*AMA1 was bound to an equilibrated glutathione-agarose column, and then His-*Et*ESP was added to the column. The proteins bound to the glutathione-agarose, and any non-specifically bound proteins were removed by elution with buffer. The proteins retained on the column were then eluted and detected with immunoblotting using anti-r*Et*AMA1 and anti-r*Et*Esp antibodies (Fig. 5). The results clearly indicated a direct interaction between the *Et*AMA1 and *Et*Esp proteins.

### *Et*Esp is not secreted from the microneme

**Fig. 6:**
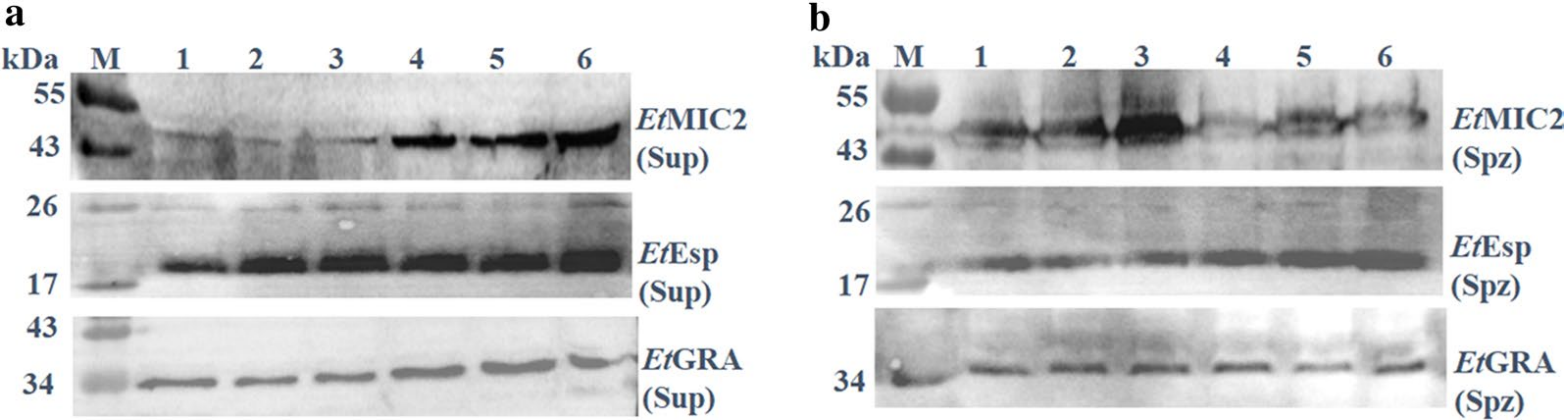
Western blotting analysis of secretion assays (supernatants and sporozoites pellet). **a** The supernatants (Sup). **b** The sporozoites pellet (Spz). Lane M: protein marker; Lanes 1–3: 5, 10 and 20 μM staurosporine dissolved in DMSO; Lanes 4–6: volumes of DMSO solvent corresponding to 5, 10 or 20 μM staurosporine

To examine the secretion of *Et*Esp, sporozoites were incubated in CM at 41 °C. The supernatant containing the excretory-secretory antigens (ESA) from the incubated sporozoites and sporozoites pellets were analyzed with western blotting. Immunoblots of the ESA samples and sporozoites were probed with an anti-r*Et*Esp antibody and showed that *Et*Esp was secreted when the sporozoites were incubated at 41 °C under 5% CO_2_ in CM. Rabbit serum raised against the micronemal protein *Et*MIC2 was used as the experimental control [27]. To demonstrate whether *Et*Esp secretion is dependent on the micronemal pathway, we added staurosporine to the CM because staurosporine is a protein kinase inhibitor known to specifically inhibit microneme secretion [28]. In the parasites treated with 5, 10 or 20 μM staurosporine, the secretion of *Et*Esp and *Et*GRA into the supernatant was not affected, but the secretion of *Et*MIC2 in supernatant was significant reduced compared with their secretion in the presence of the DMSO solvent only (Fig. 6a, b). These results show that *Et*Esp is a secreted protein, but not a micronemal protein.

### Immunolocalization of *Et*Esp at different developmental stages of *E. tenella*

**Fig. 7:**
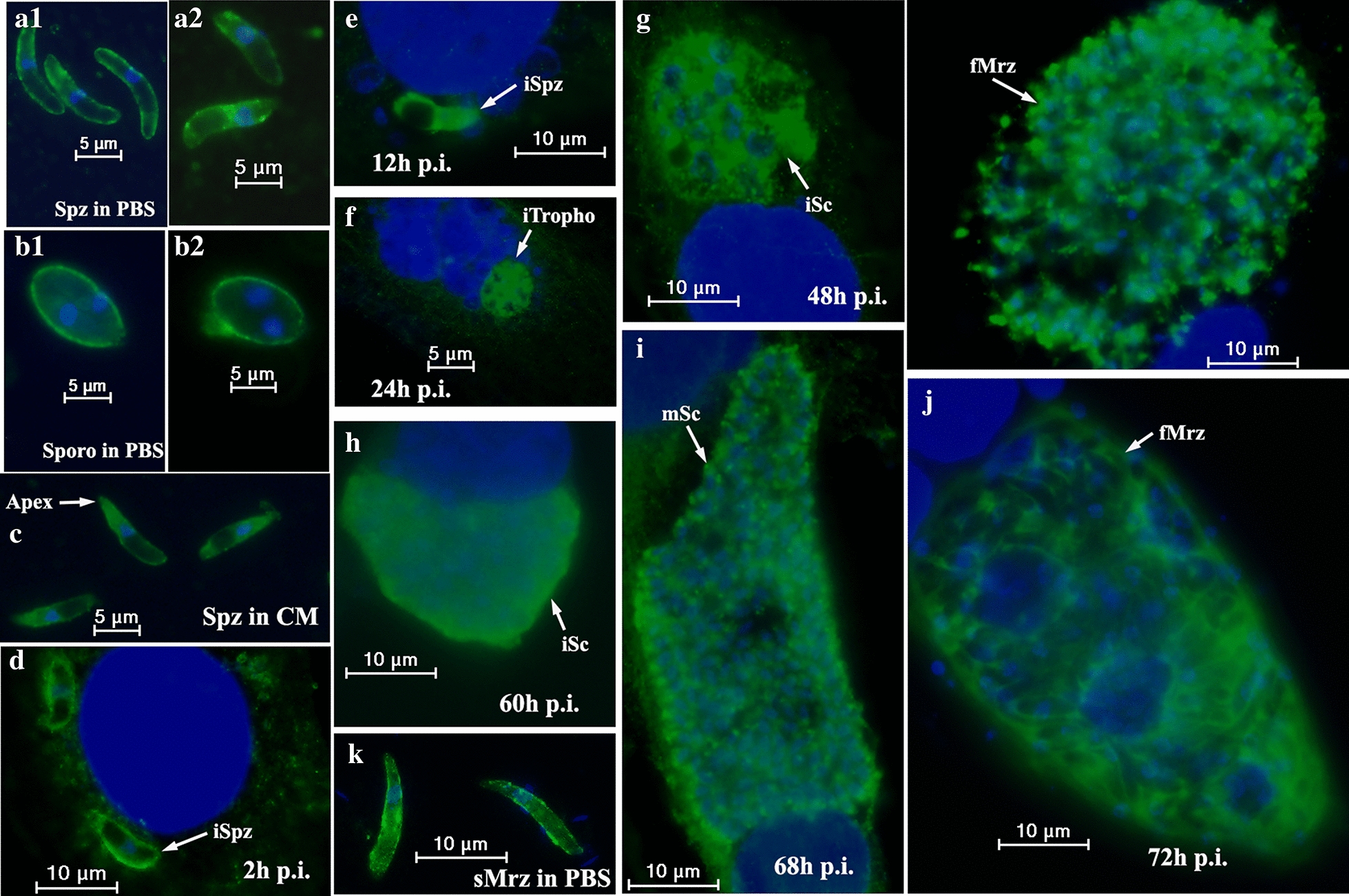
Immunofluorescent localization of *Et*Esp at different developmental stages of *E. tenella*. Parasites were immuno-stained with anti-r*Et*Esp antibody. **a** Sporozoites (Spz) were incubated in PBS (**a1** permeabilized sporozoites, **a2** non-permeabilized sporozoites). **b** Sporocysts (Sporo) were incubated in PBS (**b1** permeabilized sporocysts, **b2** non-permeabilized sporocysts). **c** Sporozoites (Spz) were incubated in complete medium (CM) for 2 h at 41 °C. **d**, **e**, intracellular sporozoites (iSpz) at 2 h and 12 h post-infection, respectively. **f** Trophozoites (iTropho) at 24 h post-infection. **g**, **h** Immature schizonts (iSc) at 48 and 60 h post-infection, respectively. **i** Mature schizonts (mSc) at 68 h post-infection. **j** First-generation merozoites (fMrz) at 72 h post-infection. **k** Second-generation merozoites (sMrz) in PBS

To investigate the localization and distribution of the *Et*Esp protein in different development stages of *E. tenella*, including sporozoites, second-generation merozoites, immature schizonts, and mature schizonts, the protein was localized with immunofluorescence *in vitro* using an antibody against r*Et*Esp. The *Et*Esp protein was mainly distributed on the surfaces of the permeabilized parasite sporozoites, sporocysts, and second-generation merozoites (Fig. 7a1, b1, k). The protein was also mainly located on the surface of non-permeabilized sporozoites and sporocysts (Fig. 7a2, b2). After incubation in CM for 2 h, the fluorescence increased and the protein was mainly localized to the anterior and surface of the parasites (Fig. 7c). *Et*Esp protein was also mainly located on the surface of the parasites 2 h after their invasion of DF-1 cells (Fig. 7d). At 12 h after the sporozoites were added to DF-1 cells, *Et*Esp also localized to the cytoplasm of the sporozoites, except for the refractile body in the posterior section of the parasites, and the intensity of *Et*Esp staining had increased (Fig. 7e). At 24–72 h post-infection, the *Et*Esp protein was uniformly distributed in trophozoites, immature schizonts, and mature schizonts, and the protein’s expression had increased (Fig. 7f–j).

### Co-localization of *Et*AMA1 and *Et*Esp

**Fig. 8:**
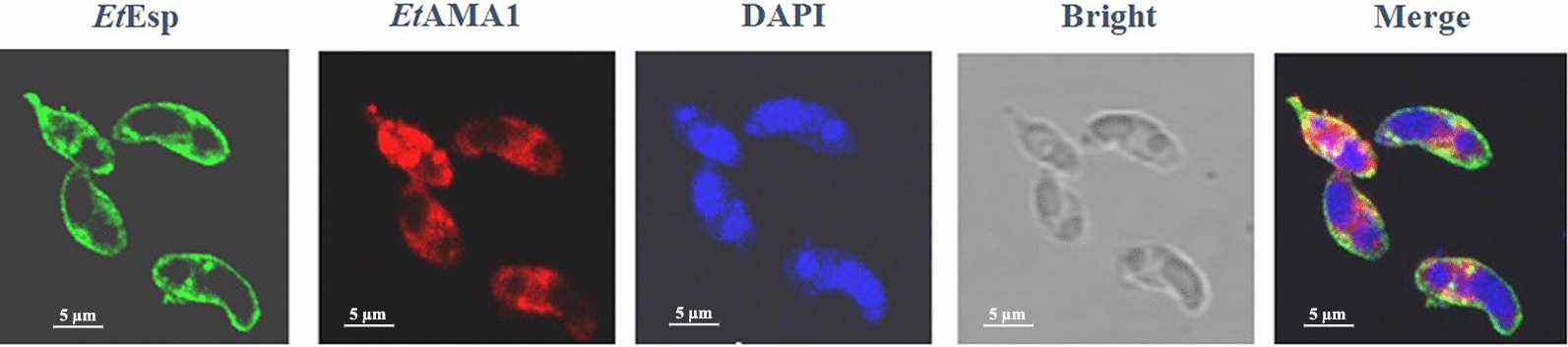
Co-localization of *Et*Esp and *Et*AMA1 in sporozoites using mouse anti-r*Et*AMA1 antibody and rabbit anti-r*Et*Esp antibody by IFA

IFAs were performed to determine the location of *Et*AMA1 and *Et*Esp. Purified sporozoites were treated with mouse anti-r*Et*AMA1 antibody and rabbit anti-r*Et*Esp antibody. The results showed *Et*Esp was mainly located on the surface of sporozoites, *Et*AMA1 was distributed throughout the cytoplasm and the membrane of sporozoites except for refractile bodies (Fig. 8).

### Anti-r*Et*Esp antibodies inhibit DF-1 cell invasion

**Fig. 9:**
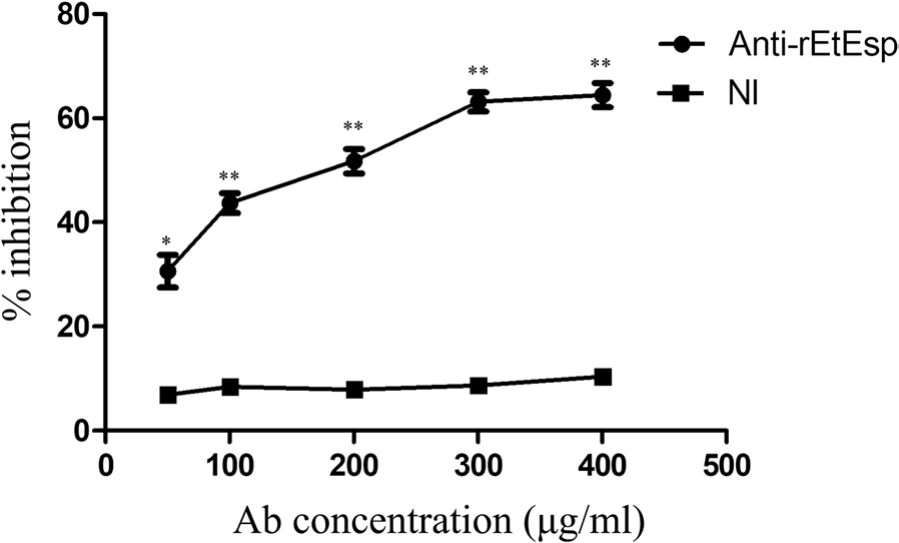
Inhibition of sporozoite invasion *in vitro*. All assays were performed in triplicate. *Abbreviations*: anti-r*Et*Esp, rabbit antiserum generated against recombinant *Et*ESP protein; NI, IgG from naïve rabbit serum. **P < 0.01 for differences between treatment with antibody against r*Et*Esp or with naïve rabbit serum with the same IgG concentration

To evaluate the effect of the *Et*Esp protein on the invasion of DF-1 cells by *E. tenella* sporozoites, an invasion inhibition assay of sporozoites was performed *in vitro*. When the sporozoites were incubated with purified anti-r*Et*Esp antibody before infection, their capacity to invade the DF-1 cells was significantly reduced. After pre-treatment with 50, 100, 200, 300 or 400 μg/ml anti-*rEt*Esp IgG antibody, their invasion of cells was highly significantly reduced compared with that of sporozoites treated with naïve rabbit IgG (negative control) (*P* < 0.01). Under these experimental conditions, an inhibition plateau of 62.9% was reached at an antibody concentration of 300 μg/ml. In a comparative analysis, the same dose of the naïve rabbit serum IgG antibody did not significantly affect invasion (Fig. 9).

## Discussion

In this study, we cloned and characterized the *E. tenella Eimeria*-specific protein, a putative *Et*AMA1-interacting protein, using a yeast two-hybrid system in our laboratory [22]. Although the yeast two-hybrid system is a widely used and powerful method for identifying the partners of proteins in regulatory complexes and in the analysis of protein-protein interactions [30], the system has several limitations, including the possibility of isolating very large numbers of clones with no biological relevance [31]. Therefore, the interaction between *Et*AMA1 and *Et*Esp required validation with an alternative technique, such as a BiFC assay or GST pull-down assay. The BiFC assay is a versatile technique for investigating protein-protein interactions in living systems, and is based on the reconstitution of a fluorescent protein *in vivo* [32]. GST pull-down is amenable to more specific investigations of protein-protein interactions *in vitro*, but relies on purified proteins that may not fully mimic the protein’s native conformation or post-translational modification, which mediate its interactions [33]. Although these assays have some advantages in identifying protein-protein interactions, each also has its drawbacks. Therefore, in many research fields, these methods are often combined to identify the interactions between two proteins [33–35]. In this study, the interaction between *Et*AMA1 and *Et*Esp was confirmed with a GST pull-down assay *in vitro* and a BiFC assay *in vivo*. These results indicated that *Et*Esp interacts with *Et*AMA1.

Proteins perform a vast number of cellular functions through their interactions with one or multiple binding partners. Moreover, many protein-protein interactions are regulated by post-transcriptional modification (e.g. phosphorylation) of the protein of interest, and these modifications are induced by exposure to certain circumstances [36]. In the present study, an amino acid sequence analysis predicted that *Et*Esp contains one N-glycosylation site, five casein kinase II phosphorylation sites, six N-myristoylation sites, one tyrosine kinase phosphorylation site and one intein DOD-type homing endonuclease domain. Inteins, also called protein introns, are parasitic genetic elements that excise themselves at the protein level by self-splicing, allowing the formation of functional, non-disrupted proteins [37]. These data suggest that its functions may be regulated by post-translational modification. We supposed that the interaction of *Et*AMA1 with *Et*Esp may be regulated by post-translational modification.

To understand the expression of *Et*Esp in the different developmental stages of the parasite, we examined its expression patterns with qPCR and western blotting. Our results indicated that *Et*Esp mRNA levels were higher in second-generation merozoites and sporozoites than in sporulated oocysts or unsporulated oocysts. But western blotting showed that the expression of *Et*Esp was higher in sporozoites than other developmental stages of *E. tenella.* Immunofluorescent localization showed that the expression of the protein increased with the development of the parasites in DF-1 cells. Previous proteomic and transcriptomic data confirm that *Et*AMA paralogues are tightly stage-regulated [38, 39]. *Et*AMA1 is a sporozoite-specific protein involved in the invasion process of sporozoites [10, 19, 38]. While another *Et*AMA1 paralogues, *Et*AMA2 is a merozoites-specific protein not involved in the parasite invasion. All these finding indicate that *E. tenella* parasites harbour stage-specific AMA proteins that could be relevant during specific phases of the parasite cycle [19]. In this study, *Et*Esp is differentially expressed during the distinct phases of the parasite life-cycle and may be very important in the invasion and development of the parasite life-cycle.

Immunofluorescent localization showed that *Et*Esp was located on the surface of the sporozoite and concentrated around the anterior of the parasite during its incubation in CM. However, the protein has no transmembrane region or glycophosphatidyl inositol (GPI)-anchor sequence, but has a signal peptide, and six N-myristoylation sites. The presence of a signal peptide is necessary for the translocation of proteins from their ribosomal sites of translation into the lumen of the endoplasmic reticulum, from where they are trafficked in the endomembrane system to their final locations within the cell or beyond [40]. We speculated that *Et*Esp also undergoes post-translational modification according to the amino acid sequence analysis, including phosphorylation, myristoylation, and glycosylation. Among these modifications, myristoylation is the key factor in the membrane localization of signal-transducing proteins [41].

Most surface antigens are involved in the invasion, pathogenesis and immune evasion of parasites. For example, in *Plasmodium*, merozoite surface proteins are critical for parasite invasion, and represent attractive targets for antibody-based therapies against clinical malaria [42]. We also found that the expression of *Et*Esp increased and that the protein mainly localized on the anterior and surface of the parasites after incubation in CM for 2 h. This suggests that the protein is involved in the sporozoite invasion of host cells. To investigate the function of *Et*Esp in the invasion process, we performed an invasion test *in vitro* and found that polyclonal rabbit anti-r*Et*Esp serum efficiently reduced the sporozoite invasion of cultured DF-1 cells. *Et*AMA1 also localized to the anterior of the sporozoites after their invasion of DF-1 cells [10]. Previous reports have shown that monospecific mouse anti-r*Et*AMA1 serum or polyclonal rabbit antiserum against r*Et*AMA1 also blocked the invasion of host cells *in vitro* [10, 19]. In the present study, *Et*Esp is involved in invasion as demonstrated by using antibodies raised against *Et*Esp *in vitro*. We tested whether *Et*Esp is an interacting protein with *Et*AMA1 by using BiFC, GST-pull down and yeast two-hybrid system. Therefore, we speculated that *Et*Esp mediates sporozoites invasion in host cells by interacting with *Et*AMA1. The exact function of *Et*Esp requires further study.

Previous studies have shown that in *T. gondii* and *Plasmodium*, AMA1 interacts directly with rhoptry neck protein 2 (RON2), which is secreted from the parasite rhoptries and specifically localizes at the moving junction. The RON2-AMA1 interaction is a critical step in the moving-junction-dependent invasion of host cells by apicomplexan parasites [43, 44]. Although the interaction between AMA1 and RON2 has not been reported in *Eimeria* spp., *E. tenella* is an apicomplexan and AMA1 is conserved in this phylum. Therefore, we inferred that *Et*AMA1 may also interact with *Et*RON2 and specifically localize to the moving junction during the invasion of host cells by *E. tenella*. In this study, we have shown that *Et*Esp is an *Et*AMA1-interacting protein, but whether it localizes to the moving junction during invasion requires further study.

## Conclusions

In this study, we have shown that *Et*Esp interacts with *Et*AMA1 using a BIFC assay *in vivo* and a GST pull-down assay *in vitro*. Using staurosporine, we showed that *Et*Esp is a secreted protein of sporozoites but not from micronemes. An invasion inhibition assay revealed that an antibody against r*Et*Esp also blocked parasite invasion of its host cells by more than 62%. These data have implications for the use of *Et*AMA1 or *Et*AMA1-interacting proteins as targets in therapeutic intervention strategies against avian coccidiosis.

## Supplementary information

**Additional file 1: Table S1.** Primer sequences used in this study.

## Data Availability

The datasets used and/or analysed during the present study are presented in the article and its additional file or are available from the corresponding author upon reasonable request.

## Abbreviations

*Et*AMA1: *Eimeria tenella* apical membrane antigen 1
*Et*Esp: *E. tenella Eimeria*-specific protein
BiFC: Bimolecular fluorescence complementation
GST pull-down: Glutathione S-transferase pull-down
RACE: Random amplification of PCR ends
UO: Unsporulated oocysts
SO: Sporulated oocysts
Spz: Sporozoites
sMrz: Second-generation merozoites
NCBI: National Center for Biotechnology Information
ORF: Open reading frame
CM: Complete medium
