# Supplementary material for: Eimeria tenella Eimeria-specific protein that interacts with apical membrane antigen 1 (EtAMA1) is involved in host cell invasion
Source: Parasit Vectors. 2020 Jul 25;13:373. doi: 10.1186/s13071-020-04229-5 (PMC7382093; doi:10.1186/s13071-020-04229-5)
Supplement: Supplementary file 1 — Additional file 1: Table S1. Primer sequences used in this study. [file 13071_2020_4229_MOESM1_ESM.doc]

**Additional file 1: Table S1.** Primers sequence used in this study

| Primer name | Primer sequence |
| --- | --- |
| GS5P (5' Primer) | 5'-ATGGTCTCGGGCCAGTTCTCGTTCA -3' |
| GS5N (5' Nested Primer) | 5'-GGAGATGAGACCCAGGCGGATGAAA -3' |
| *Et*Esp-UP | 5'-GCGGATCCATGAAGGGCCTGTTCTTCACCGTCG-3' |
| *Et*Esp-LP | 5'-GCCTCGAGCGAATCTACTTCAAGAAAAGCCACG-3' |
| *Et*Esp-SP | 5'-CCCCGACTACCTCAAGTTCCTCAGC -3' |
| *Et*Esp-AP | 5'-TGGGTCCGTCTCCCCCTCCTTGGTG -3' |
| 18S-SP | 5'-TGTAGTGGAGTCTTGGTGATTC-3' |
| 18S-AP  Bf*Et*ESp-UP  Bf*Et*ESp-LP  Bf*Et*AMA1-UP  Bf*Et*AMA1-LP | 5'-CCTGCTGCCTTCCTTAGATG-3'  5'-GCGAATTCGGGCCACCATGAAGGGCCTGTTCTT-3'  5'-GCAGATCTGCTGCTCGCGTTGCCAGCAGAT -3'  5'-GCGAATTCGGGCCACCATGCAGCCGCCCTAT-3'  5'-GCCTCGAGGGTATTCCTGGTCCAG-3' |
